# Generation of liver mesenchyme and ductal cell organoid co-culture using cell self-aggregation and droplet microfluidics

**DOI:** 10.1016/j.xpro.2023.102333

**Published:** 2023-06-03

**Authors:** Anna M. Dowbaj, Timo N. Kohler, Lucía Cordero-Espinoza, Florian Hollfelder, Meritxell Huch

**Affiliations:** 1 Max Planck Institute of Molecular Cell Biology and Genetics, Dresden 01307, Germany; 2 Wellcome Trust–Medical Research Council Stem Cell Institute Cambridge, Cambridge CB2 1QR, UK; 3 Department of Biochemistry, University of Cambridge, Cambridge CB2 1GA, UK; 4 Wellcome Trust/Cancer Research UK Gurdon Institute, Cambridge CB2 1QN, UK; 5 Department of Physiology, Development and Neuroscience, University of Cambridge, Cambridge CB2 3DY, UK

**Keywords:** Cell Biology, Stem Cells, Organoids

## Abstract

Within the peri-portal region of the adult liver, portal fibroblasts exist in close proximity to epithelial ductal/cholangiocyte cells. However, the cellular interactions between them are poorly understood. Here, we provide two co-culture techniques to incorporate liver portal mesenchyme into ductal cell organoids, which recapitulate aspects of their cellular interactions *in vitro*. We integrate several techniques from mesenchyme isolation and expansion to co-culture by microfluidic cell co-encapsulation or 2D-Matrigel layer. The protocol is easily adaptable to other cells from other organs.

For complete information on the generation and use of this protocol, please refer to Cordero-Espinoza et al*.*^1^

## Highlights

• Isolation, sorting, and *in vitro* expansion of primary liver portal mesenchyme
• Production of microfluidic PDMS chips
• Droplet microfluidic method for primary liver mesenchyme/ductal cell organoid co-culture
• Cell self-aggregation method for primary liver mesenchyme/ductal cell organoid co-culture

## Abstract (editor highlights)

Publisher’s note: Undertaking any experimental protocol requires adherence to local institutional guidelines for laboratory safety and ethics.

## Graphical abstract

**Figure:**
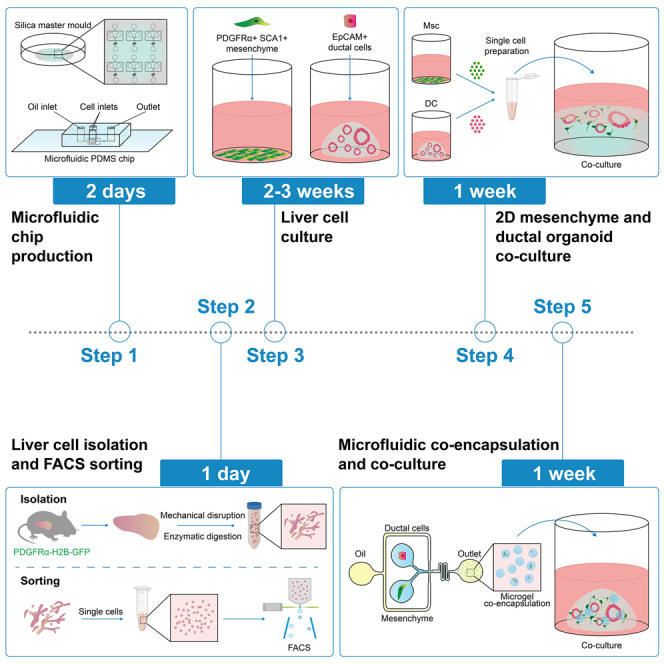

## Before you begin

In Cordero-Espinoza et al.*,*^1^ we reported that when primary liver mesenchyme is co-cultured with liver ductal epithelium in 3D classical Matrigel dome, the cells fail to establish cell-cell interactions. The cells segregate in the well with the mesenchyme attaching to the plate bottom and the ductal cells forming organoids inside of the Matrigel (for reference see Figure S4M from ref. ^1^). Therefore, we developed two co-culture methods: a microfluidics -based method and a 2D Matrigel layer co-culture method, to enable the cell-cell interactions between both populations *in vitro*. The microfluidics approach facilitates the cell-cell interactions recapitulating the 3D architecture (See Figure 5 and Figure S5 from ref. ^1^), while the 2D-co-culture method allows more control of the ratios between cell types (See Figure 6 and Figure S6 from ref. ^1^) as well as it is amenable to chemical and siRNA manipulation (See Figure 7 from ref. ^1^).

The protocol below explains the specific steps for co-culturing primary liver ductal organoids with primary liver mesenchymal cells, mainly portal fibroblasts. Both, mesenchymal cells and ductal cells can be co-cultured directly after isolation or after several sub-cultivations (2–5 passages for portal mesenchyme and any passage for ductal organoids). Notably, at the passages tested, the cells do not present any change on their phenotype following culture (for details see Figure 4D-E and Figures S4D–S4F from ref. ^1^). Workflows involving microfluidic cell encapsulation and co-culture protocols in Matrigel are broadly applicable to other cell types.

Cells were isolated from PDGFRα-H2B-GFP mice (B6.129S4-*Pdgfra*^tm11(EGFP)Sor^/J)^2^ which can be crossed with mTmG mice (*Gt(ROSA)26Sor*^*tm4(ACTBtdTomato,-EGFP)Luo*^/J)^3^ for the easier double fluorescence labeling of the mesenchyme. For the PDGFRα-H2B-GFP line, these mice will only be bred as heterozygous, because homozygous mice are not viable. A wild-type mouse is also necessary as a control for sorting in each experiment. The detailed protocol is applicable to adult mice of all ages although the mice used for the experiments presented here are adults between 8-12 weeks of age. From one mouse, the total cell yield is 10 000–40 000 of all cells achieved with this digestion method. Typical total number of cells from this kind of preparation ranges from 10–80 million cells. Typical viability after sorting is >90%.

In addition, this protocol describes production of microfluidic chips made of polydimethylsiloxane (PDMS) by soft lithography. The design of the microfluidic chip (triple inlet flow focusing device) is available as a CAD file from https://openwetware.org/wiki/DropBase:Droplet_3_inlets. Microfluidic chips were produced based on master molds obtained via soft lithography with an SU-8 photoresist on a silica substrate, as described elsewhere,^4^^,^^5^ in polydimethylsiloxane (PDMS).

### Microfluidic chip production

**Timing: 24 h**

**Figure 1:**
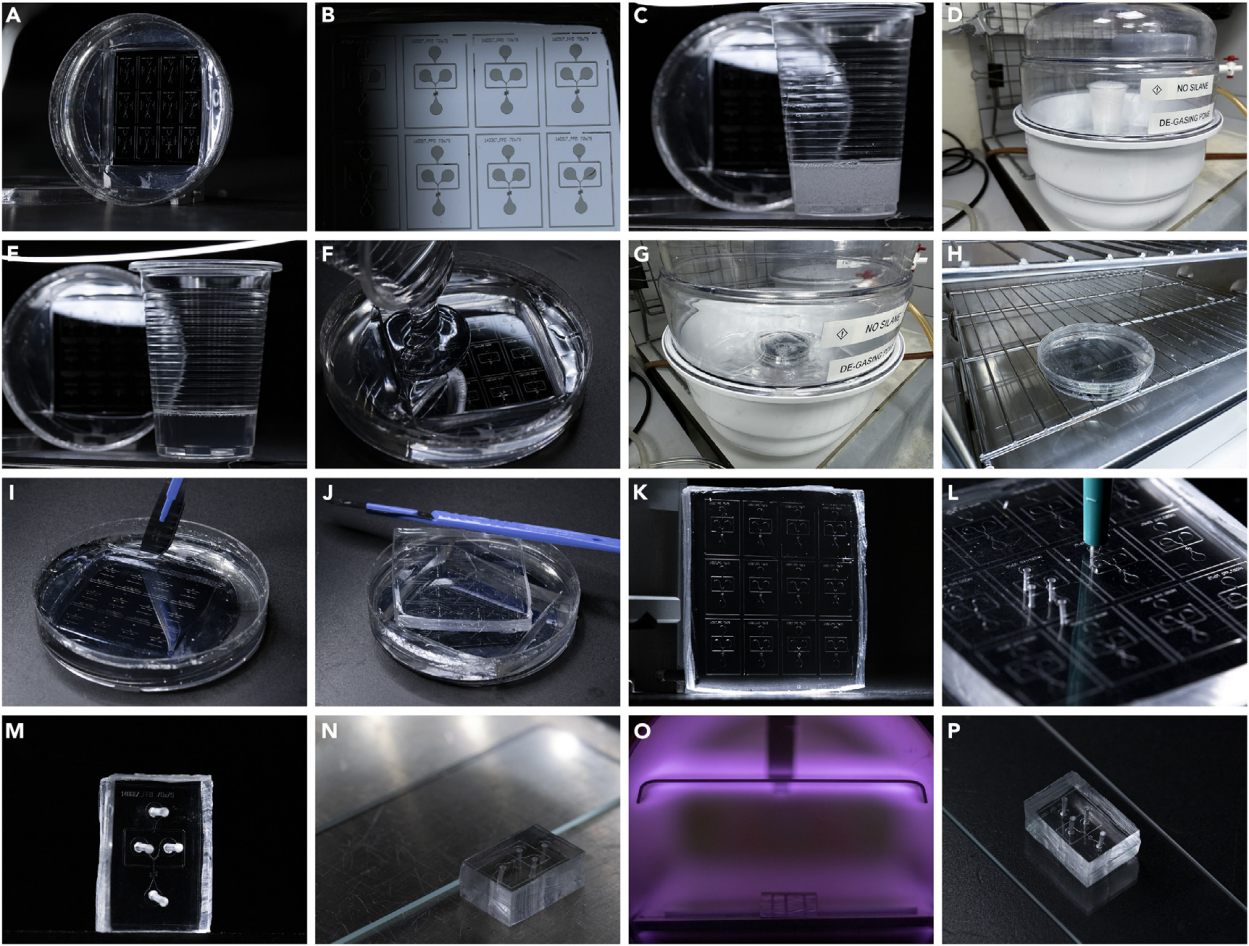
Microfluidic chip production (A) Master mold silica wafer before addition of PDMS. (B) Detailed view of silica wafer. (C) SYLGARD™ 184 Silicone Elastomer and curing agent (10:1 ratio) mixed in a plastic cup. (D) De-gassing of SYLGARD™ 184 Silicone Elastomer mixture. (E) Air bubble-free SYLGARD™ 184 Silicone Elastomer mixture. (F) Pouring SYLGARD™ 184 Silicone Elastomer mixture onto the silica wafer. (G) De-gassing of SYLGARD™ 184 Silicone Elastomer mixture on the chip. (H) Curing of SYLGARD™ 184 Silicone Elastomer mixture at 65°C 12–18 h overnight. (I–K) Use a scalpel to carefully remove the polymerized SYLGARD™ 184 Silicone Elastomer (PDMS). (L) Use a 1 mm disposable biopsy needle to create the tubing inlets and outlet of the PDMS chip. (M) Single PDMS chip ready for plasma treatment. (N) Place cleaned microscopy slide and PDMS chip (channels facing upwards) in the plasma cleaner. (O) Oxygen plasma:treat the PDMS chips and microscopy slides for 12 s with oxygen plasma. (P) Directly afterward, flip the PDMS chips by 180° (now with the channels facing downwards again) and gently press them onto the microscopy cover slides.

For a detailed illustration of the chip production please refer to Figure 1.

1. Prepare silicone elastomer for subsequent chip production (Figures 1A–1C).
  a. Mix SYLGARD™ 184 Silicone Elastomer and curing reagent (10:1 ratio) in a plastic cup and mix thoroughly (Figure 1C).
  b. De-gas the SYLGARD™ 184 Silicone Elastomer mixture in a desiccator under vacuum until no air bubbles raise to the surface (Figures 1D and 1E).
  c. Pour the de-gassed SYLGARD™ 184 Silicone Elastomer mixture onto the SU-8-derived silica master mold (Figure 1F).
  d. De-gas the freshly poured SYLGARD™ 184 Silicone Elastomer again in a desiccator under vacuum (Figure 1G).

**CRITICAL:** Ensure that the SYLGARD™ 184 Silicone Elastomer mixture is devoid of bubbles as entrapped air will cause undesirable cavity formation during polymerization.

2. Cure the poured SYLGARD™ 184 Silicone Elastomer mixture at 65°C for 12–18 h overnight (Figure 1H).

***Note:*** SYLGARD™ 184 Silicone Elastomer can also be cured at 19°C–25°C (room temperature). However, this process will take at least 48 h.

3. After polymerization, use a scalpel to carefully cut the SYLGARD™ 184 Silicone Elastomer (PDMS) (Figures 1I–1K).
  a. Use a 1 mm disposable biopsy needle to punch the tubing inlet and outlets of the PDMS chip (Figures 1L and 1M).
  b. Wash the PDMS chip briefly (5–20 s) by placing it in a 50 mL test tube filled with 2-propanol and vortex it for 10 s.
  c. Dry the PDMS with compressed air.
  d. Clean microscopy slides (as many as PDMS chips need to be produced) by removing any dust particles with scotch tape.

**CRITICAL:** The inlets/outlets should fit with the desired tubing’s outer diameter, where the tubing can be fitted with ease whilst sitting there tightly (i.e. resistant to a mild pulling force).

***Note:*** Be gentle when cutting the PDMS in order not to damage the underlying silica master mold.

***Note:*** Make sure to remove any residual PDMS from the puncture holes and clean the microscopy slides thoroughly. Any remaining PDMS or dust particles could clog the chip during cell encapsulation and disrupt the experiment.

4. Place the microscopy slides and PDMS chips in the plasma cleaner (Femto plasma system; Diener Electronic, Germany) (Figure 1N).

**CRITICAL:** Check that the PDMS chips are placed in the correct orientation. The channels must face upwards to be exposed to the plasma.

5. Prepare a 1% (v/v) solution of trichloro(1H,1H,2H,2H-perfluorooctyl)silane (PFOCTS) in HFE-7500.
  a. Fill a 1 mL plastic syringe with the 1% PFOCTS solution and attach a needle with 5 cm long tubing.
6. Oxygen plasma: treat the PDMS chips and microscopy slides for 12 s with oxygen plasma (Figure 1O).
7. Directly afterward, flip the PDMS chips by 180 ° (now with the channels facing downwards again) and gently press them onto the microscopy cover slides (Figure 1P).
8. Silane treatment: flush channels with the freshly prepared and filtered solution of 1% (v/v) PFOCTS in HFE-7500.
9. Incubate the PFOCTS-filled PDMS chips for 15 min at 65°C (in an oven or a heating plate).

**CRITICAL:** The placing of PDMS onto the coverslips and the subsequent silane treatment must be done immediately after the plasma treatment.

**Pause point:** After silane treatment, the PDMS chips can be stored for up to 6 months under sterile conditions.

### Prepare reagents for cell isolation, sorting, and seeding

**Timing: 15 min**

10. Before starting the cell isolation, prepare all the media in advance.
  a. Media can be prepared ahead of time and stored in the fridge for up to 1 month (Refer to the “materials and equipment” section for media composition tables).
  b. Prepare 1% FBS DMEM media.
11. Prepare Advanced DMEM /F12 +++ media.
12. Prepare collagenase-dispase II solution (Refer to the “materials and equipment” section for media composition tables).
  a. Prepare the collagenase-dispase II solution immediately before use.
  b. Weigh collagenase and dispase immediately before adding 1% FBS DMEM.
  c. Warm up the solution to 37°C.

**CRITICAL:** This solution cannot be stored for longer than the time of the isolation experiment, as the enzymes lose activity over time when in solution.

13. During the isolation incubation times, prepare the staining solutions for flow cytometry.
  a. For each liver, prepare 1.5 mL staining solution with all antibodies, with 15 μL of each antibody per 1.5 mL of media.
  b. For the control WT liver, add 1 μL of single antibody solution per polypropylene tube per single color control with 100 μL of cell suspension.
  c. Prepare collection test tubes with 300 μL Advanced DMEM /F12 +++ media supplemented with ROCK inhibitor (10 μM final concentration).
14. Thaw matrigel on ice at 4°C 12–18 h (overnight).

## Key resources table

**Table:** 

REAGENT or RESOURCE	SOURCE	IDENTIFIER
**Antibodies**		

Rat anti-Ly-6A/E (Sca-1) monoclonal (Clone D7), Super Bright 436 (dilution 1:100)	Thermo Fisher Scientific	Cat# 62-5981-82; RRID: AB_2637287
Rat anti- CD326 (EpCAM) monoclonal (Clone G8.8), APC (dilution 1:100)	Thermo Fisher Scientific	Cat# 17-5791-80; RRID: AB_2734965
Rat anti-CD31 monoclonal (Clone 390), PE-Cy7 (dilution 1:100)	BD Biosciences	Cat# 561410; RRID: AB_10612003
Rat anti-CD45 monoclonal (Clone 30-F11), PE-Cy7 (dilution 1:100)	BD Biosciences	Cat# 552848; RRID: AB_394489
Rat anti-CD11b monoclonal (Clone M1/70), PE-Cy7 (dilution 1:100)	BD Biosciences	Cat# 552850; RRID: AB_394491

**Chemicals, peptides, and recombinant proteins**		

Collagenase from *Clostridium histolyticum*	Merck/Sigma	Cat# C9407
Dispase II	Thermo Fisher Scientific	Cat# 17105-041
DNAse (Deoxyribonuclease I from bovine pancreas)	Merck/Sigma	Cat# DN25
Fetal bovine serum	Merck/Sigma	Cat# F7524
Advanced DMEM/F-12	Thermo Fisher Scientific	Cat# 12634010
DMEM, high glucose, GlutaMAX Supplement, pyruvate	Thermo Fisher Scientific	Cat# 31966021
HEPES (1M)	Thermo Fisher Scientific	Cat# 15630056
Penicillin/Streptomycin	Thermo Fisher Scientific	Cat# 15140-122
GlutaMAX supplement	Thermo Fisher Scientific	Cat# 35050-068
TrypLE Express Enzyme (1×), phenol red	Thermo Fisher Scientific	Cat# 12605010
TrypLE Select Enzyme (10×), no phenol red	Thermo Fisher Scientific	Cat# A1217701
B27-Supplement, serum free	Thermo Fisher Scientific	Cat# 17504-044
N-acetylcysteine (NAC)	Merck/Sigma	Cat# A9165
[Leu15]-Gastrin I Human	Merck/Sigma	Cat# G9145; CAS: 39024-57-2
Mouse EGF Recombinant Protein	Thermo Fisher Scientific	Cat# PMG8041
Recombinant Human FGF-10	Peprotech	Cat# 100-26
Recombinant Human HGF (Insect derived	Peprotech	Cat# 100-39
Nicotinamide	Merck/Sigma	Cat# N0636
Rspondin 1 (RSPO1) conditioned medium	Home-made as in Broutier et al., 2016 Nature protocols^6^	N/A
WNT3a conditioned medium	Home-made as in Broutier et al., 2016 Nature protocols^6^	N/A
ROCK inhibitor - Y-27632 dihydrochloride	Merck/Sigma	Cat# Y0503; CAS: 129830-38-2
Matrigel Growth Factor Reduced (GFR) Basement Membrane Matrix, Phenol Red-free	Corning	Cat# 356231
HFE-7500 3M (TM) Novec (TM) Engineered fluid	Fluorochem	Cat# 051243; CAS: 297730-93-9
Pico-Surf (5% (w/w) in Novec 7500)	Sphere Fluidics	Cat# C022
SeaPrep Agarose	Lonza	Cat# 50302
1H,1H,2H,2H-Perfluoro-1-octanol (PFO)	Merck/Sigma	Cat# 370533; CAS: 647-42-7
Sylgard 184	Farnell	Cat#101697

**Experimental models: Cell lines**		

Mouse: liver mesenchyme SCA1^+^ PDGFRα^+^	Cordero-Espinoza et al., 2021 Cell Stem Cell^1^	N/A

**Experimental models: Organisms/strains**		

Mouse: mTmG [*Gt(ROSA)26Sor*^*tm4(ACTBtdTomato,-EGFP)Luo*^/J] Any age or gender. We preferably use animals <12 weeks but is not required	The Jackson Laboratory	RRID: IMSR_JAX:007576
Mouse: PDGFRα-H2B-GFP [B6.129S4-*Pdgfra*^tm11 (EGFP)Sor^/J] Any age or gender. We preferably use animals <12 weeks but is not required	The Jackson Laboratory	Hamilton et al., 2003^2^; RRID: IMSR_JAX:007669

**Software and algorithms**		

FlowJo	FlowJo	https://www.flowjo.com/
QmixElements (pump software)	Cetoni	https://cetoni.com/

**Other**		

SGE gas tight 2.5 mL glass syringe, Luer lock	Merck	Cat# 509493
SGE gas tight 100 μL glass syringe, Luer lock	Merck	Cat# 509469
BD Microlance 3 needles (0.4 × 19mm)	BD	Cat# 302200
LDPE Tubing (inner ⌀ 0.4 mm; outer ⌀ 0.8 mm)	Reichelt Chemietechnik	Cat# 28464
Nemesys low pressure syringe pump	Cetoni	Cat# NEM-B101-03 A
Base 120 pump unit	Cetoni	Cat# NEM-B100-01 F
Inverted light microscope	Leica	https://www.leica-microsystems.com/products/light-microscopes/p/leica-dm-il-led/
High speed camera	PCO	https://www.pco.de/highspeed-cameras/pcodimax-cs4/

## Materials and equipment

**Table:** 

1% FBS DMEM	Final concentration	Amount
DMEM	N/A	490 mL
Pen/Strep	1%	5 mL
FBS	1%	5 mL
**Total**	**N/A**	**500 mL**

Store up to 1 month at 4°C.

**Table:** 

Advanced DMEM /F12 +++	Final concentration	Amount
Advanced DMEM/ F12	N/A	489 mL
Pen/Strep	1%	1 mL
GlutaMAX	1%	5 mL
HEPES	10 mM	5 mL of 100× stock
**Total**	**N/A**	**500 mL**

Store up to 1 month at 4°C.

**Table:** 

Collagenase-dispase II∗	Final concentration	Amount
1% FBS DMEM	N/A	60 mL
Collagenase	0.0125% (mg/mL)	8.4 mg
Dispase II	0.0125% (mg/mL)	8.4 mg
DNAse	0.1 mg/mL	6 mg
**Total**	**N/A**	**60 mL**

∗numbers for 1 mouse liver; scale accordingly

**CRITICAL:** prepare immediately before the experiment, cannot be stored and reused in suspension.

**Table:** 

Mesenchymal media (MM)	Final concentration	Amount
Advanced DMEM /F12 +++	N/A	14 mL
WNT3a Conditioned media	30%	6 mL
B27	1%	0.4 mL of 50× stock
N2	1%	0.2 mL of 100× stock
N-acetylcysteine	1.25 mM	25 μL of 500mM stock
Rock kinase inhibitor Y27632	10 μM	20 μL of 10mM stock
**Total**	**N/A**	**20 mL**

Store up to 2 weeks at 4°C.

**Table:** 

Expansion media (EM)	Final concentration	Amount
Advanced DMEM /F12 +++	N/A	9.5 mL
RSPO1 Conditioned media	5%	0.5 mL
B27	1%	0.2 mL of 50× stock
N2	1%	0.1 mL of 100× stock
N- acetylcysteine	1.25 mM	12.5 μL of 500 mM stock
Gastrin	10 nM	1 μL of 100 μM stock
Mouse EGF	50 ng/mL	1 μL of 500 μg/mL stock
FGF10	100 ng/mL	10 μL of 100 μg/mL stock
HGF	50 ng/mL	5 μL of 100 μg/mL stock
Nicotinamide	10 mM	100 μL of 1M stock
**Total**	**N/A**	**10 mL**

Store up to 2 weeks at 4°C.

**Table:** 

Antibody staining solution (per 1 liver)	Final concentration	Amount
**1% FBS DMEM**	N/A	1.5 mL
Anti-EPCAM-APC	3 μg (stock 0.2 mg/mL)	15 μL
Anti-CD45-PECy7	3 μg (stock 0.2 mg/mL)	15 μL
Anti-CD31-PECy7	3 μg (stock 0.2 mg/mL)	15 μL
Anti-CD11b-PECy7	3 μg (stock 0.2 mg/mL)	15 μL
Anti-SCA1(D7)-SuperBright436	3 μg (stock 0.2 mg/mL)	15 μL
**Total**	**N/A**	**1.5 mL**

**CRITICAL:** prepare immediately before the experiment, cannot be stored and reused in suspension.

**Table:** 

Silane treatment solution	Final concentration	Amount
HFE-7500 Novec™	99.0%	990 μL
Trichloro(1H,1H,2H,2H-perfluorooctyl)silane (PFOCTS)	1.0%	10 μL
**Total**	**N/A**	**1 mL**

**CRITICAL:** prepare immediately before the experiment, cannot be stored and reused in suspension.

**Table:** 

Agarose solution	Final concentration	Amount
SeaPrep Agarose	3%	15 mg
PBS	N/A	500 μL
**Total**	**N/A**	**500 μL**

**CRITICAL:** prepare immediately before the experiment, it is not recommended to keep dissolved.

**Table:** 

0.3% suftactant in oil	Final concentration	Amount
HFE-7500 3M™ Novec oil	N/A	3 mL
5% Pico-Surf1	0.3%	0.18 μL
**Total**	**N/A**	**3.18 mL**

Store up to 1 year at 19°C–25°C.

## Step-by-step method details

### Isolation of liver mesenchyme and ductal cells

**Timing: 5–6 h**

Isolation of the primary cells from the liver tissue using mechanical disruption and enzymatic digestion. This step produces a single cell suspension of various liver cells (adapted from ref. ^6^).

1. Euthanize the mice and dissect the liver; all standard euthanasia methods yield similar results for isolation.
  a. Wash the livers several times to remove as much blood as possible; stop when PBS is mostly clear.
    ***Note:*** No gentle washing necessary – vigorous washing is allowed at this step.
  b. Transfer livers to a petri dish, remove gallbladder and any peritoneal fat or non-liver pieces of tissue.
  c. Remove all PBS or transfer to a new petri dish without PBS and chop the liver into pieces with scissors or with 1 razor blade; make sure pieces are very small (∼ 1–2 mm length and width).
  d. Transfer the pieces to a new tube containing 10 mL of PBS.

2. Let the pieces settle down by gravity or, alternatively, spin at 120 Relative Centrifugal Force (RCF)/2 min. Discard the supernatant to remove as much blood as possible.
3. Wash the pellet with 40 mL of PBS by inverting the tube several times
4. Repeat step 2.
5. Add 10 mL of collagenase/dispase media mix (top up to approx. 12.5 mL, adjust it to the tissue pellet size). In that step the addition of DNAse is optional.
6. Incubate 10 min at 37°C in a water bath.
7. Pipette up and down with 10 mL serological pipette.
8. Spin at 120 RCF/5 min and discard the supernatant to remove as many blood cells as possible.
9. Resuspend in 25 mL of collagenase/dispase media mix supplemented with DNAse.
10. Incubate 90 min at 37°C in a horizontal shaker at 130 RPM.
11. Check digestion under a brightfield microscope; some ducts and some parenchyma should be present in the mixture.

**Figure 2:**
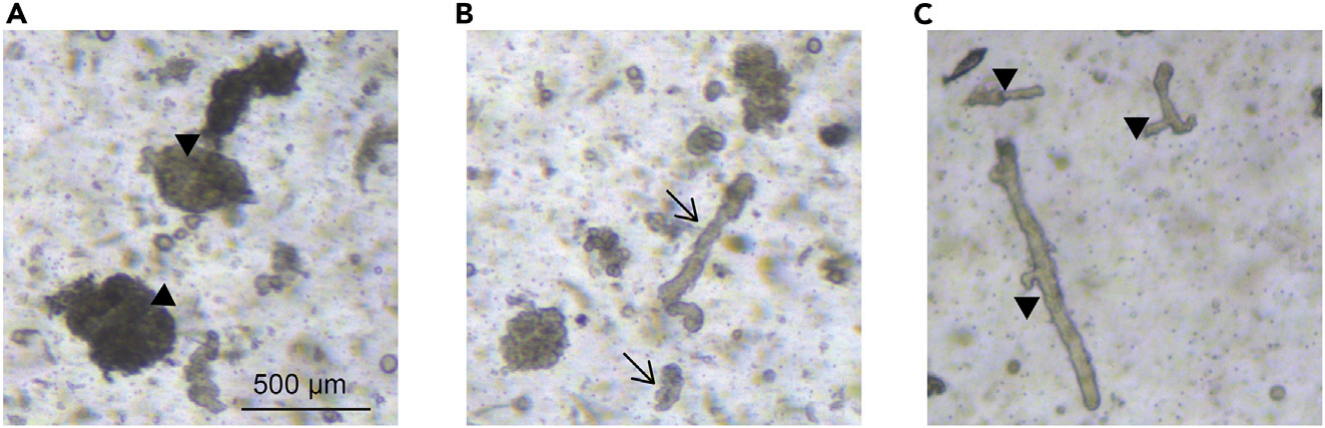
Example of digested ducts (A–C) (A) Under-digested ducts (arrowheads) – a lot of parenchyma is still present; (B) Correctly digested ducts (arrows) – some parenchyma is still present, preserving the ductal-mesenchymal connection but other clear ducts are also present; (C) Over-digested ducts (arrowheads) – no mesenchyme or parenchyma present on the ducts – this can decrease the mesenchymal yield, as they are already detached into the solution and directly exposed to the digestion mix as single cells. Scale bar, 500 μm.

**CRITICAL:** In contrast to ductal isolation from previous publication,^6^ make sure isolated ducts have some parenchyma present on them and do not appear completely cleared of parenchyma (Figure 2).

12. Take off ∼15 mL of the supernatant after the remaining pieces of tissue settle on the bottom and proceed to step 19.
13. With the reminder of tissue suspension repeat from step 7 as follows;
14. Pipette up and down with 10 mL serological pipette
15. Spin at 120 RCF/5 min and remove the supernatant.
16. Resuspend in 25 mL of collagenase/dispase media solution supplemented with DNAse.
17. Incubate 90 min at 37°C shaker horizontally with 130 RPM.
18. Check digestion under a brightfield microscope; ducts and some parenchyma should be present in the mixture (Figure 2).

***Note:*** Ducts are branched structures, which become visible upon tissue digestion (Figure 2A - ducts under-digested, the parenchyma appears around giving the ducts rough edges and ball-like appearance Figure 2C – over-digestion, ducts without any parenchyma). To enrich for the portal mesenchyme around the ducts, digest the tissue as shown in Figure 2B – where ducts appear mostly smooth-edged, but still retaining some of the parenchyma that contains the portal mesenchyme.

19. Spin at 200 RCF/5 min and remove the supernatant.
20. Wash pellet with 40 mL of Advanced DMEM/F12+++ media.
21. Spin at 200 RCF /5 min and remove the supernatant.
22. Resuspend in 5 mL 5×TrypLE with glass Pasteur pipette (make up 5×TrypLE by mixing 1×TrypLE with 10×TrypLE in 1:1 ratio).
23. Incubate 10 min at 37°C in a water bath.
24. In the meantime, prepare the pre-conjugated antibodies for flow cytometry sorting.

***Note:*** The sorting described below was performed on different instruments, namely MoFlo Legacy, Astrios (Beckman Coulter), BD FACSAria (BD Biosciences) or SH800S (SONY) cell sorters. For these instruments, we recommend the following color combinations: Super Bright 436, GFP, APC, PE-Cy7, or similar fluorophores. The use of more colors is possible, but depending on the FACS sorter machine availability. PE can easily be included in this color combination, for e.g. as a dead cell dye.

***Note:*** In case of cross talk between PE and PE-Cy7 fluorophore (instrument-dependent), we recommend the Brilliant Violet 650 conjugates to the same monoclonal antibodies (excitation: laser line 405 nm, emission: similar to PE-Cy7).

25. Resuspend cells again with glass Pasteur pipette.
26. Check for the presence of single cell suspension under a brightfield microscope; if 80% of the cells are not single cells repeat step 23 until a single cell suspension where > 80% of cells are single cells is obtained.
27. Add 5 mL of 1% FBS DMEM media.
28. Pass the mix through 40 μm strainer.
29. Pass the mix again through a new 40 μm strainer. At this step the estimated total cell yield is variable between 10–80 million cells, with above 90% of viability.
30. Top up with 40 mL of 1% FBS DMEM media.
31. Spin at 500 RCF/3 min and discard the supernatant.
32. Resuspend the cell pellet in 1 mL of 2% FBS DMEM media or relevant amount of blocking solution.
33. Proceed to step 34 for sorting.

***Note:*** The addition of DNAse in step 9 and step 16 vastly improves FACS cell sorting as the presence of DNA fragments keeps cells attached during sorting, which can clog the instrument nozzle and reduces the yield of single cells.

### Flow cytometric sorting of liver portal mesenchyme and ductal cells

**Timing: 2 h**

Isolation of desired cell populations (mesenchyme, ductal cells) from the single cell heterogeneous preparation of the liver. The purified cell populations can be used for further experiments directly, or expanded in culture.

**Figure 3:**
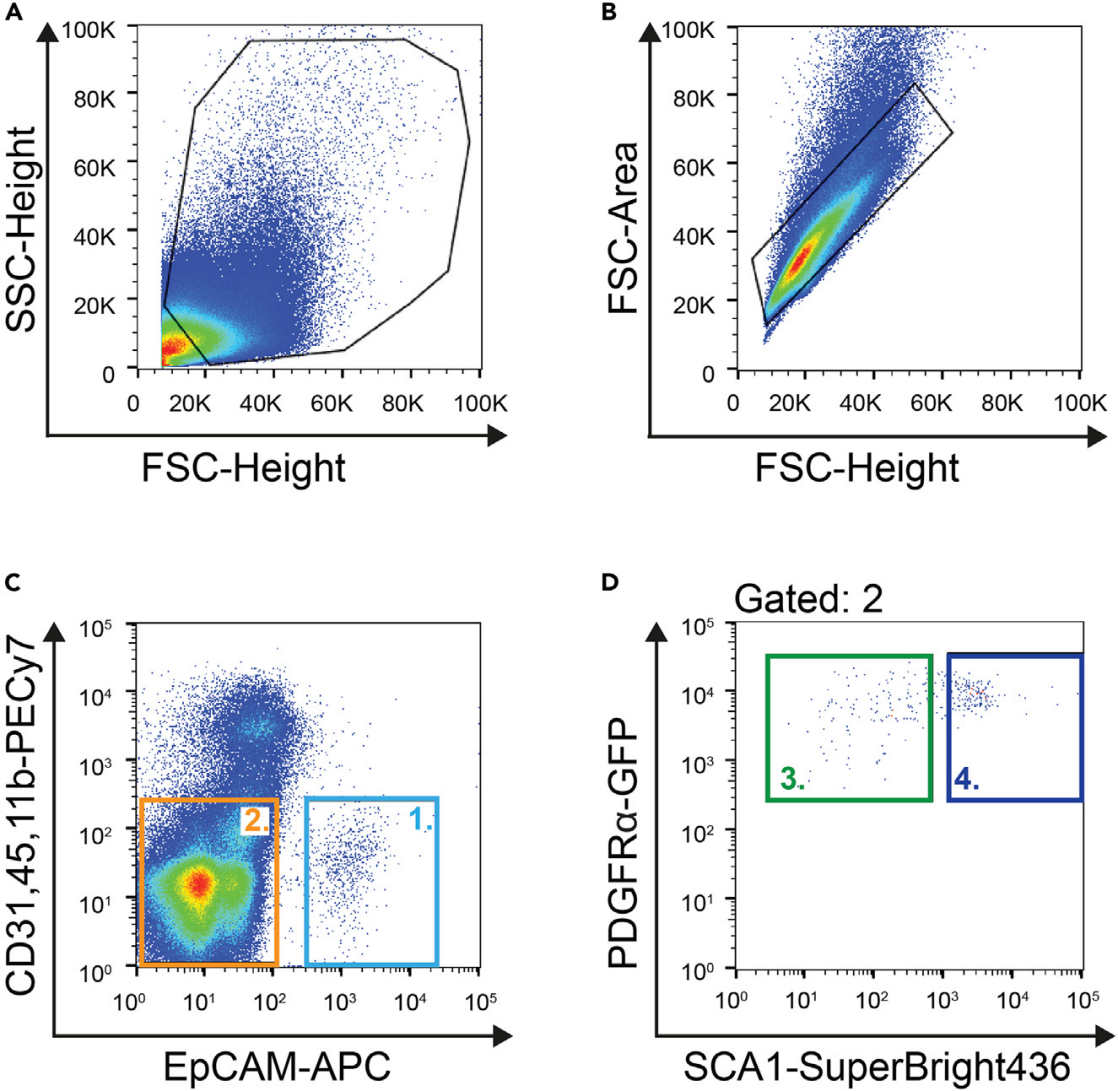
Flow cytometry cell sorting and gating (A–D) (A) Gating on liver cells – the liver prep has a lot of cell debris, which should be excluded with this gate; (B) Gating on single cells; (C) Gating on ductal cells (gate 1) and stromal cells (gate 2, excluding EpCAM positives and blood/immune positives); (D) From gate 2, gating on PDGFRα-GFP^+^ and SCA1^+^ (gate 4) or SCA1^-^ (gate 3) mesenchyme.
42. At the sorter, gate first on the cells of interest using side-scatter and forward-scatter height plot; try to remove as much debris as possible, which appears as a bright cloud shown in red in Figure 3A. There is a lot of debris in this type of liver digestion.
43. Remove cell doublets by gating as shown in Figure 3B, using a forward-scatter area *vs.* height plot.
44. Gate on PECy7 negatives (this step removes any remaining blood, immune and endothelium fractions) and APC-EpCAM negatives (this step removes liver ductal cells) – the double negatives are shown in Figure 3C.
45. When sorting ductal cells from the same preparation, gate and sort the EpCAM^+^ cells (Figure 3C, gate 1). At this step, a typical expected yield is 50 000–100 000 ductal cells/liver, with >90% viability.
46. Gate on SCA1-SB436^+^ and PDGFRα-GFP^+^ double positives (you can also include SCA1^-^ if needed), as shown in Figure 3D.
47. Collect cells in 300 μL of Advanced DMEM/ F12 +++ media. At this step, a typical expected yield is 10 000–40 000 SCA1^+^ PDGFRα-GFP^+^ mesenchymal cells/liver, with >90% viability.
48. After sorting, spin cells at 500 RCF/5 min.
49. Resuspend cells in 200 μL of MM and plate in 1 well of 96 well tissue culture-treated plates at a ratio of up to 10 000 cells/well. Proceed to step 50 for culturing.

***Note:*** If the PDGFRα-H2B-GFP mouse model is not available, antibody staining against murine PDGFRα protein is possible.

***Note:*** Here, gating on EpCAM positives allows isolation of the ductal cells as well using the same antibody staining strategy.

***Note:*** Certain FACS-sorting machines have issues with crosstalk between PE (tdTomato channel) and PE-Cy7, as the PE-Cy7 composite fluorophore also has a small emission peak at the spectral height of PE; additionally, PE-Cy7 can be degraded and therefore provide additional fluorescence confounding signal of PE; to avoid this, we recommend the Brilliant Violet 650 conjugates to the same monoclonal antibodies (excitation: laser line 405 nm, emission: similar to PE-Cy7).

34. Add 4 mL of 2% FBS DMEM and transfer to FACS-tubes (e.g., polypropylene 5 mL tube). Incubate cells on ice for 10 min (blocking step).
35. Take out samples for staining controls: 100 μL sample.
36. Centrifuge samples 500 RCF/3 min and remove the supernatant.
37. Resuspend cell pellet in 1.5 mL antibody solution and incubate for 30 min on ice.
38. For staining control add 1 μL of each relevant antibody to previously taken 100 μL sample cell suspension aliquots, and incubate for 30 min on ice.
39. Spin at 500 RCF / 3min, discard the supernatant and wash the pellet with 4 mL 1% FBS DMEM media.
40. Spin at 500 RCF/3 min, remove the supernatant and resuspend in 1 mL 1% FBS DMEM media (controls with 350 μL); if needed alter this volume depending on the sorter capacity for cell density as well as the size of your cell pellet.
41. Go to the FACS sorter (Figure 3).

**CRITICAL:** The 96-well plate has to be tissue culture treated for attaching cells, e.g. flat bottom Nunclon Delta-Treated plates from ThermoFisher.

### Liver mesenchyme *in vitro* culture

**Timing: 2 weeks**

Expansion and passaging of liver portal fibroblast population in standard cell culture on plastic dishes.

**Figure 4:**
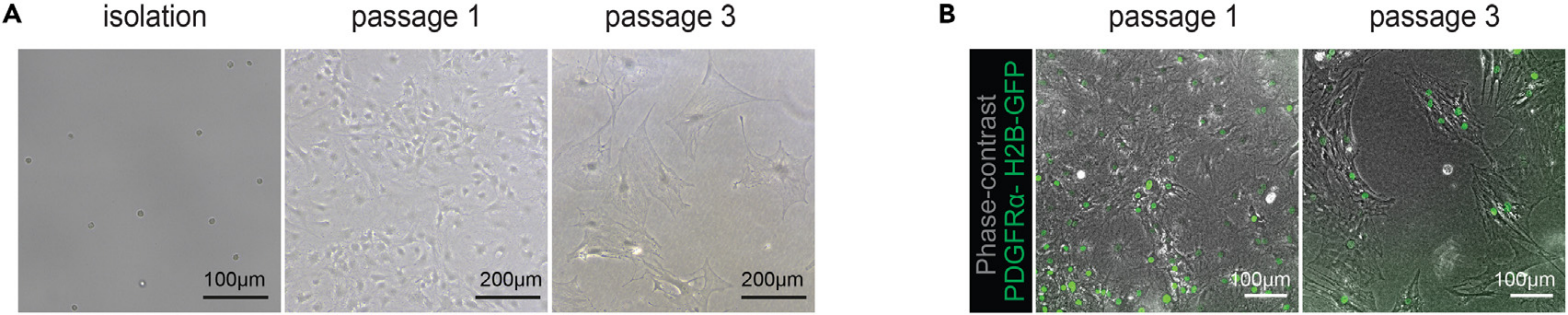
Portal Mesenchyme culture in 2D Note the change in cell morphology between passages. (A) Representative images of isolated PDGFRα-GFP^+^ / SCA1^+^ mesenchymal cells after FACS sorting (brightfield), at passage 1 and at passage 3 (phase-contrast). Scale bar, 100 μm or 200 μm. (B) Representative images of mesenchymal cells at passage 1 and passage 3, grown on plastic in MM medium. Phase-contrast (gray) and PDGFRα-GFP expression (green). Scale bar, 100 μm. For more pictures of mesenchyme passages, refer to Figure S4D in ref. ^1^

***Note:*** We do not recommend passaging mesenchymal cells described here for more than 2 passages, as we observed change in appearance (Figures 4A and 4B, passage 3 – more spread cells) as well as an increase in the expression of activated fibroblast marker αSMA, as noted in our previous manuscript (see ref. ^1^ Figure S4F).

50. Expand sorted mesenchymal cells (PDGFRα^+^ SCA1^+^) up to passage 2 (Figure 4).
51. After sorting and plating the cells in the adequate tissue culture plate incubate at 37°C 5% CO_2_; do not change media for at least 3 days – these cells need some time to attach.
52. Change media every 3–4 days.
53. Split cells when 80%–90% confluent, up to 1:4 ratio.
54. To split, wash the well once or twice with sterile PBS and aspirate.
55. Add 200 μL of pre-warmed 1×TrypLE (for a 48-well plate, scale up or down proportionally).
56. Incubate for 5 min at 37°C 5% CO_2_ in the incubator.
57. After 5 min check the cells under a brightfield microscope, they should have started rounding and detaching.
58. Pipette the cells up and down with the p200 pipette (using the same 200 μL of TrypLE) to finish detaching the cells from the plate.
59. Transfer cell suspension into a 15 mL falcon containing 10 mL of 1% FBS DMEM media.
60. Wash the well once with 200 μL of 1% FBS DMEM to remove any remaining detached cells and add them to the falcon tube.
61. Spin at 300 RCF/5 min and remove the supernatant.
62. Resuspend in MM media.
63. Use minimal media volumes per plate: 96-wells -200 μL, 48-wells – 300 μL, 24 wells – 500 μL.
64. For further media changes use MM media.

### Matrigel 2D layer co-culture of liver mesenchyme and ductal progenitor cells

**Timing: 1 h**

Co-culture method description, which allows combination of liver mesenchyme and ductal organoids in a layer of Matrigel. This co-culture model allows for direct control of respective number of cell-cell contacts, which scale proportionally to the number of cells added (i.e., the cell-cell ratio).

**CRITICAL:** Matrigel will solidify at 19°C–25°C, so work quickly and keep the basement matrix cold throughout the process.

***Note:*** In the classic matrigel dome 3D culture, the 2 cell types co-cultured together segregate, with the mesenchymal cells preferentially attaching to the plate bottom and the ductal cells forming ductal organoids without any mesenchymal contact. For more information, please refer to Figure S4M in ref. ^1^.

***Note:*** The expanded mesenchyme can be used up to passage 2, and ductal organoids until passage 10. Later passages of both cell types have successfully been used to make co-cultures, but we do not recommend this for the mesenchyme, as it shows signs of fibroblast-like activation in prolonged culture.

***Note:*** For simplicity, in this manuscript we present an experiment where the number of ratios tested ranges from 0.1:1 to 1:1. However, the ratio number can be changed to any desired ratio just by adjusting the ratio of both populations. For more co-culture ratios, refer to Figure 6 and Figure S6 in ref. ^1^.

**Figure 5:**
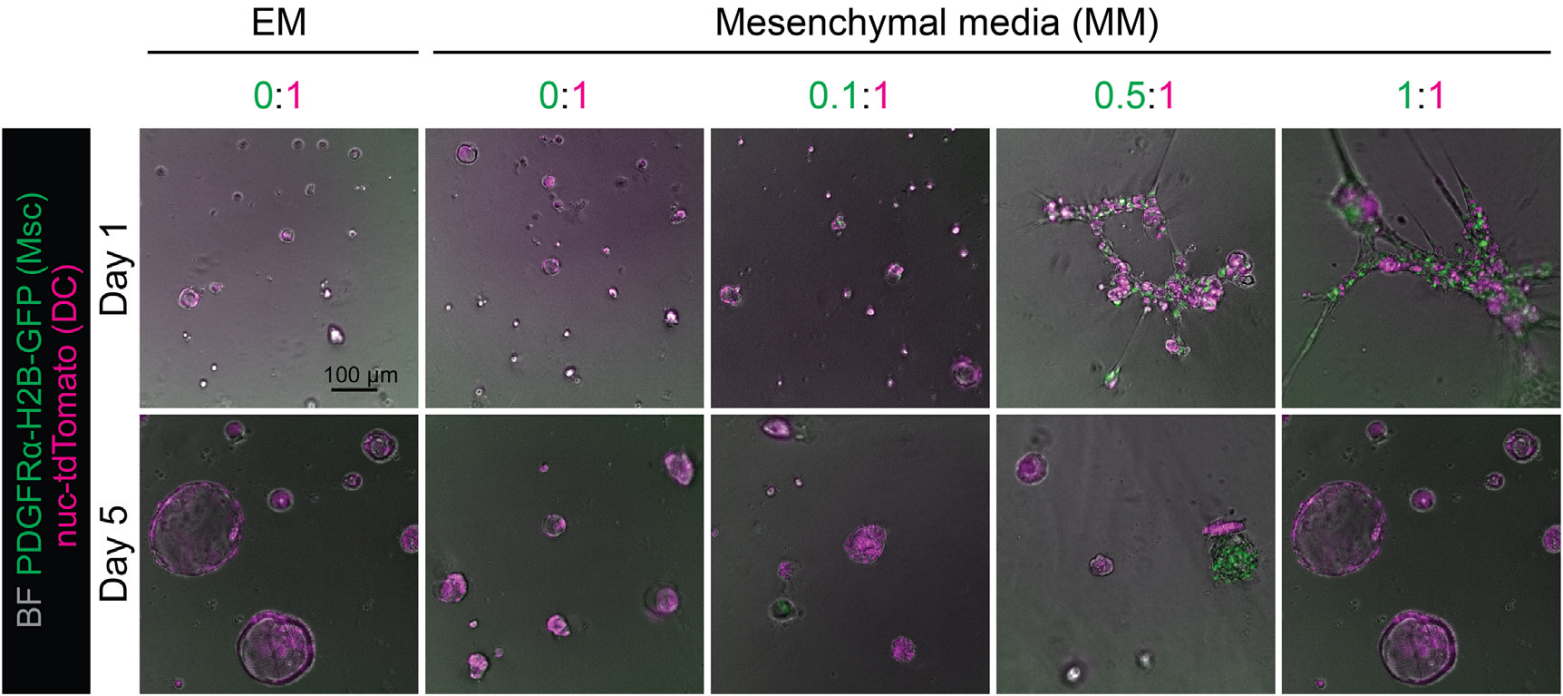
Co-culture of mesenchymal and ductal cells on a Matrigel layer Representative images of mesenchymal cells (Msc, green) cultured at different ratios with ductal cells (DC, magenta) (Msc:DC ratio); Top row: day 1; bottom row, day 5; expansion media (EM) control is shown for comparison to mesenchymal media (MM). For more co-culture ratios, refer to Figure 6 and Figure S6 in ref. ^1^ Scale bar, 100 μm.

65. Use expanded or sorted cells from steps 50 or 48, respectively.
66. Dissociate mesenchyme to single cells, following protocol as for splitting (step 53).
67. Collect ductal cell organoids grown from sorted cells in step 45 and passaged in a 3D Matrigel dome overlayed with expansion media (EM) (for more detail refer to^6^) by scraping their culture Matrigel bubble with p1000 pipette tip and placing it in 2 mL of Advanced DMEM /F12 +++ media in a 15 mL falcon tube.
68. Pipette the media up and down with p1000 or a narrowed glass Pasteur pipette.
69. Top up with Advanced DMEM/ F12 +++ media to 10 mL and spin at 200 RCF/5 min and remove the supernatant.
70. Resuspend the cells in 100 μL of Advanced DMEM/ F12 +++ media (adjust depending on the pellet size).
71. Dissociate ductal cell organoids to single cells by incubating them 5–10 min with 1 mL of pre-warmed 1×TrypLE at 37°C.
72. Top up with Advanced DMEM/ F12 +++ media to 10 mL and spin at 500 RCF/5 min and remove the supernatant.
73. Resuspend the cells in 100–500 μL of media (adjust depending on the pellet size).
74. Count single cell solution cell density for both cell types.
75. The expected number of ductal and mesenchymal cells depends on their passage number. Ductal cells have the capacity to expand long-term, therefore increasing the number of passages will yield the desired number. On average, 1 confluent well of a 24 well plate yields 50 000–100 000 cells. Mesenchymal cells have less expansion capacity, which decreases with increasing passages. On average 1 confluent well of a 24 well plate yields 20 000–25 000 cells.
76. Mix selected number of cells, always adjusting the mesenchyme ratio to 5000 ductal cells, in a test tube.
77. For e.g., for 1:1 ratio of the cells, use 5000 ductal cells and 5000 mesenchymal cells.
78. Mesenchyme to ductal cells can be mixed at any ratios, for e.g., 0:1, 0.1:1, 0.2:1, 0.5:1, 1:1, 2:1 and 5:1.
79. After mixing, centrifuge cells at 300 RCF/5 min.
80. Before seeding of the Matrigel-layer co-cultures, coat the 96-well plate wells with 30 μL of 100% Matrigel during cell centrifugation step:
  a. Pipette 30 μL of 100% Matrigel per well on 96-well plate (at 19°C–25°C).
  b. Allow Matrigel to pre-solidify for minimum of 5 and maximum of 10 min.
81. Resuspend the cells in 200 μL of media.
82. Seed on top of a 2D-layer of semi-solidified Matrigel (100%) covering the bottom of a 96-well plate.
83. Monitor the growth of the culture over the next 7–10 days (Figure 5), changing the media every 3 days.

### Microfluidic co-encapsulation of liver mesenchyme and ductal cells

**Timing: 2 h**

Second co-culture method description, which allows combination of liver mesenchyme and ductal organoids in an agarose microgels, and subsequent culture in a 3D matrigel dome. The advantage of using this co-culture method is the better recapitulation of the periportal tract *in vitro*, however this method does not allow for the control of respective cell numbers in the structure.

84. Prepare a 3% low-melting agarose solution for microfluidic cell co-encapsulation.
  a. Prepare a 3% (weight/volume) agarose solution in a test tube by weighing e.g., 15 mg agarose for 500 μL PBS.
  b. To dissolve the agarose, spread the measured-out agarose powder on tube’s walls and add an appropriate amount of PBS to ensure homogeneous distribution of the powder in PBS.
  c. Melt the agarose at 75°C in an orbital thermomixer for 30 min while shaking at 400 RPM.
  d. Once agarose is melted, cool the agarose solution to 37°C in an orbital thermomixer.

**CRITICAL:** Low-melting agarose must be used for microfluidic cell encapsulation. Other agarose preparations will solidify when cooled to 37°C.

**CRITICAL:** Agarose must be kept in the thermomixer at 37°C. Cooling it outside of a thermomixer may cause it to cool too quickly, which might cause it to solidify.

***Alternatives:*** In this protocol SeaPrep® ultra-low melting agarose (LONZA, #50302) is used. However, a variety of different low-melting agarose products are available from different suppliers. If alternatives are being used, the resulting gels may have different biophysical properties.

***Note:*** The tubing recommended here fits with 1 mm inlets/outlets for the microfluidic device used in the “Before you begin” step 3a. If necessary, tubing diameter can be adjusted depending on the size of the biopsy needle used to generate the inlets and outlets.

***Note:*** The expanded mesenchyme can be used up to passage 2, and ductal organoids until passage 10. Later passages of both cell types have successfully been used to make co-cultures, but we do not recommend this especially for the mesenchyme, as it shows signs of fibroblast-like activation in prolonged culture.

**Pause point:** Agarose can be kept at 37°C for several hours. At this point the microfluidic rig can be set up and cells can be prepared for encapsulation.

85. Prepare the oil + surfactant solution.
  a. Use HFE-7500 3M™ Novec™ (Fluorochem, # 051243) engineered fluid as the carrier oil phase.
  b. Use Pico-Surf1 surfactant (5%; Sphere Fluidics, #C022) in the above-mentioned carrier oil diluted to 0.3% to stabilize the agarose-in-oil emulsion during encapsulation.
  c. If surfactant appears milky, filter it though a 0.22 μm filter.
86. Prepare the oil (carrier phase) syringe.
  a. Prepare tubing long enough to reach from the syringe (pumps) to the chip.
  b. Use tweezers to attach the tubing to the needle.
  c. Fill the oil syringe (2.5 mL, Luer lock glass syringe) with the oil+surfactant solution (step 85: 0.3% Pico-Surf1 in HFE-7500 3M™ Novec™).
  d. Attach the tubing and needle to the syringe and push the air out, so that the whole tubing is filled with oil.
  e. Place the syringe in the pump and attach the tubing to the oil inlet of the PDMS chip.

87. Prepare cells for encapsulation.
  a. Prepare single cell suspensions of ductal and mesenchymal cells in two different tubes.
    ***Note:*** for detailed description of preparing a single cell suspension, please refer to step 54 of this protocol for mesenchyme, and step 67 for ductal cells.
  b. Pass ductal cell single cell suspension through a 40 μm strainer and subsequently collect at 500 RCF for 5 min.
  c. Mesenchymal cell suspension does not need to be strained, but if strained use also 40 μm strainer; collect cells at 500 RCF for 5 min.
  d. Resuspend each of cell types in 50 μL of MM media at a concentration of 0.5–1 × 10^6^ cells/mL.
  e. Keep cells on ice until mixing with agarose.
  f. Warm up the cell suspension by placing the cells at 37°C or by holding the tube in your hand before adding the agarose from step 84.
  g. Slowly add 50 μL of agarose solution to the cells (1:1 ratio) and carefully mix the suspension by pipetting up and down without creating air bubbles.
  h. The cells are now ready to be loaded into the syringes. Proceed to step 88.
    **CRITICAL:** To achieve the mesenchyme cell concentrations specified above, isolation from several mice is often necessary. A pilot experiment is suggested, to assess how well the primary mesenchyme expands in the *in vitro* culture.

88. Prepare the cell-laden (aqueous phase) syringes (Figure 6).
  a. Cut 2 sets of tubing and attach each to a separate needle (Figure 6A).
  b. To see the contents of the tubing better and to avoid losing the tube content, make a loop at the end of the tubing and hold it in your hand so that the tube opening is facing upwards (Figure 6B).
  c. Fill each syringe with 100 μL of HFE-7500 and attach the needle to the tubing. Make sure that the needle reservoir is also filled with HFE-7500 (Figure 6C).
  d. Push out the HFE-7500 with the syringe, leaving the oil only in the tubing (Figure 6D).
  e. Create a small air bubble at the end of the tubing by gently pulling the syringe plunger backwards (Figure 6E). The air bubble will allow monitoring of how far the tubing has been filled with agarose/cell suspension mixture.
  f. Place the end of the tubing in the agarose/cell suspension mixture and gently fill the tubing by moving the plunger slowly backwards (Figure 6F).
  g. Stop filling the tubing just before the air bubble enters the needle.
  h. Stop filling the tubing before you completely run out of cell-agarose mixture. Avoid air bubbles.
  i. Connect the tubing to the correct inlets on the PDMS chip.

**Figure 6:**
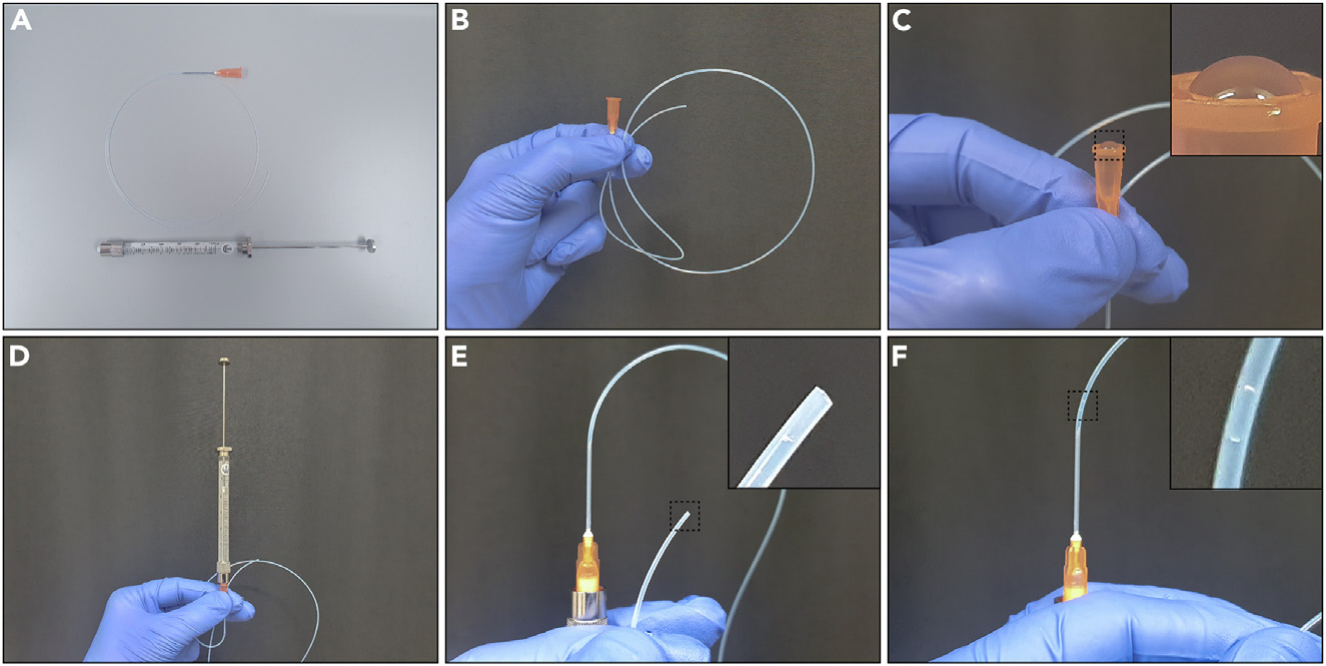
Loading of the agarose into cell syringe (A) Pre-fill the glass syringe with HFE-7000. (B) Connect the tubing with the needle. (C) Fill the needle reservoir with HFE-7500. (D) Connect the syringe with the needle and tubing, and push the HFE-7500 out of the syringe. (E) Pull the plunger back until a small air bubble appears at the end of the tubing. (F) Fill the tubing with the agarose/cell mixture using the air bubble as an indicator; stop just before the air bubble reaches the needle.
89. Add tubing for the outlet exiting the chip, and put a 1.5mL test tube on ice to collect the agarose droplets emerging from the tubing – there, they will solidify there and become microgels.
90. Perform microfluidic encapsulation (Figure 7).
  a. Switch on all the equipment (computer, pumps, microscope and camera, Figure 7A).
  b. In the pump software, select the correct syringe for each pump.
  c. Select the correct flow rate for your experiments; here we used 3 μL/min for the cell syringes and 30 μL/min for the oil syringe (Figure 7B).
  d. Modify the flow rates to change droplet size and droplet forming speed in your experiments.
  e. Run the experiment.
  f. During the run, watch out for blockages, do not leave the equipment unattended while encapsulating.
  g. Collect the cell-laden agarose droplets gels in a test tube on ice for solidification and formation of microgels
  h. Stop all equipment once the syringes are empty of the cell-agarose solutions; the oil syringe with the oil-surfactant can be reused for the next encapsulation if it is not empty.

**Figure 7:**
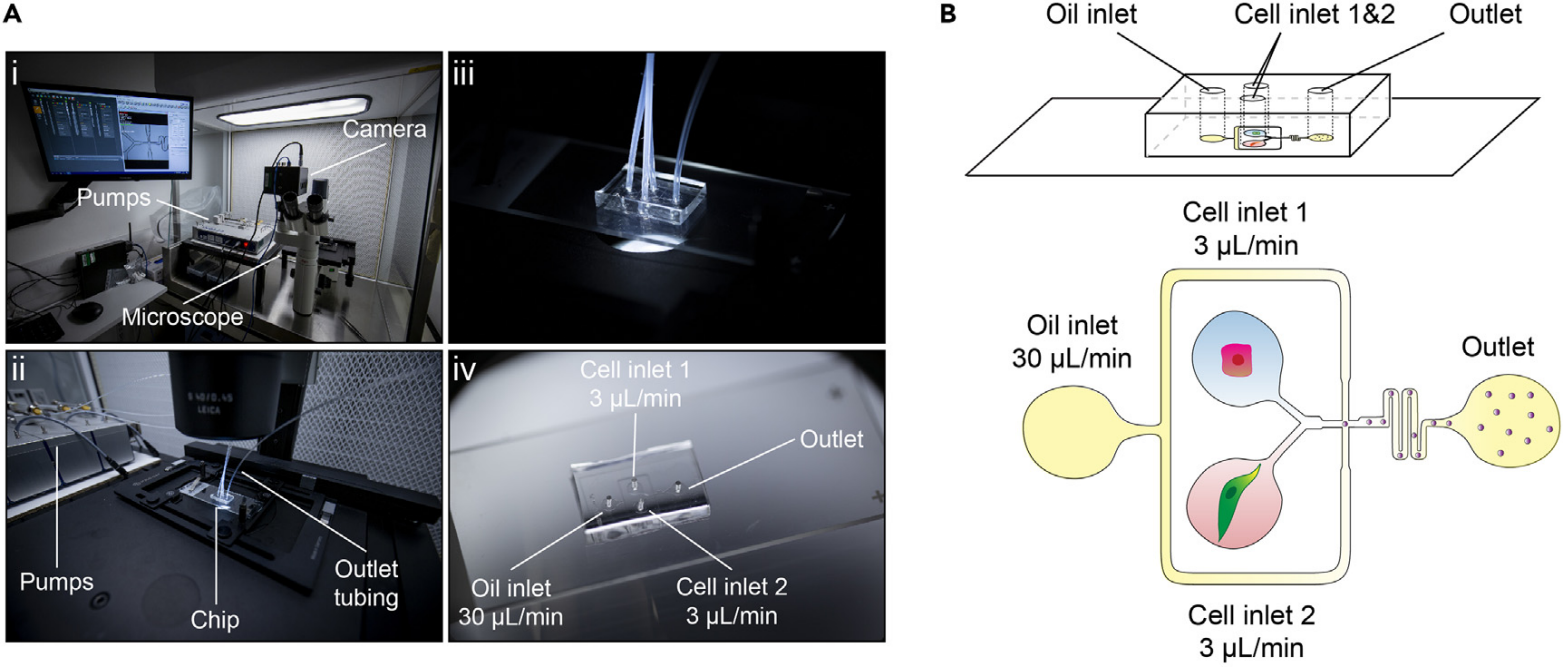
Microfluidics rig set up Set-up of the microfluidic encapsulation rig. (A) Photographic illustration of the microfluidic rig for co-encapsulation. (i) Sterile encapsulation environment including precision pumps, a high-speed camera and light microscope. (ii) The microfluidic chip connected to the pumps, ready for cell encapsulation. (iii) Close-up view of the connected microfluidic chip. iv) Disconnected chip for easier view of the inlets and outlets for the tubing. (B) Schematic illustration of the microfluidic co-encapsulation chip with labels designating inlets, outlets and flow rates used.

***Note:*** Depending on microfluidic chip design, other flow rates may be equally suitable and can be experimentally optimized. Optimal flow rates are influenced by the viscosity of the oil and the hydrogel precursor used necessitating empirical adjustment.

**Figure 8:**
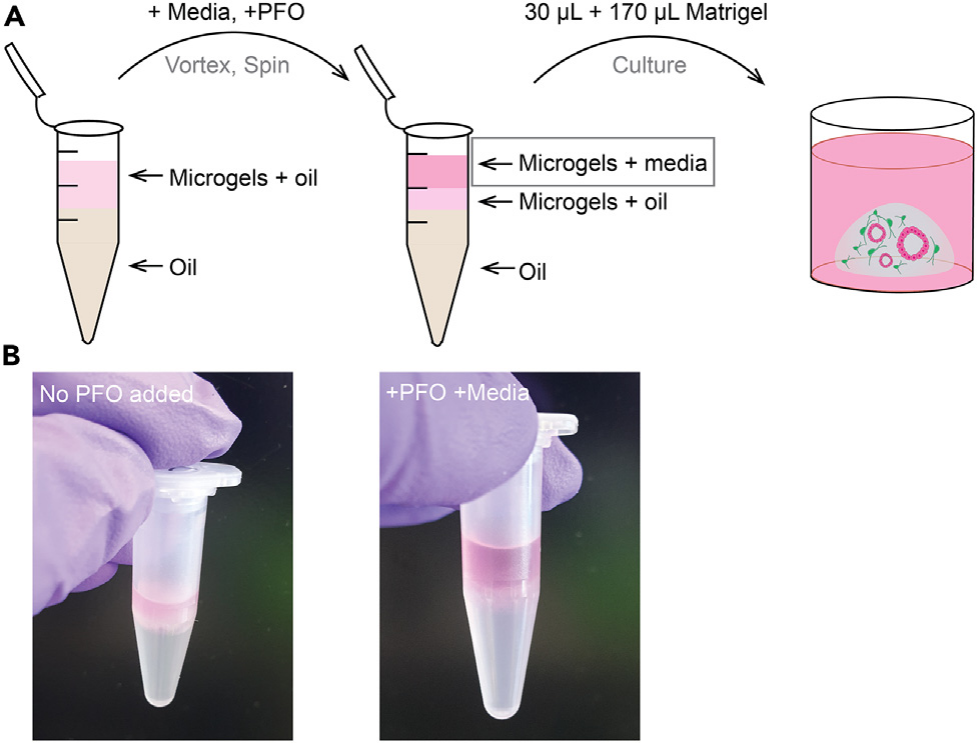
De-emulsification of encapsulated agarose-cell microgels (A) Schematic of liquid layers before and after the adition of 1H,1H,2H,2H-Perfluioro-1-Octanol (PFO); the fraction “microgels + media” should be taken for further seeding in Matrigel. (B) Photographs of the test tubes before and after the addition of PFO for the de-emulsification procedure.
  b. Remove as much oil as possible without touching the cell phase with a pipette. It is better to leave some oil rather than disrupt the microgel-laden phase.
  c. Add 200 μL of MM media to the microgels collection tube.
    **CRITICAL:** Do not pipette up and down, just add media on top of the microgels phase.
  d. Add 45 μL of 1H,1H,2H,2H-Perfluoro-1-octanol (PFO) to the microgels collection tube.
    **CRITICAL:** Do not pipette up and down, just add on top of media and microgels.
  e. Vortex for 5 s, spin briefly (5–20 s) in a microcentrifuge and take the cell suspension phase (Figure 8B).

**Figure 9:**
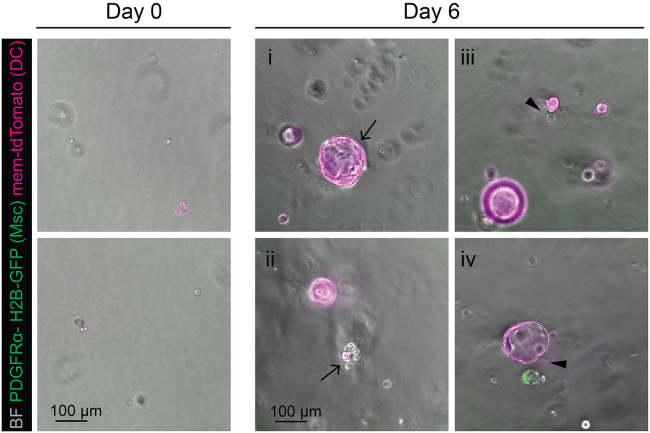
Co-culture of mesenchyme and ductal cells after microfluidic co-encapsulation Representative images of encapsulated cells embedded in Matrigel at day 0 and day 6 of culture. Representative of organoid structures where contact between ductal cells (red) and portal mesenchyme (green) is established (i, ii, arrows), as well as ductal cell organoid structures with mesenchyme growing in the vicinity but where contact has not been established (iii, iv, arrowheads). BF, bright field. Scale bar, 100 μm.

91. De-emulsify the microgels.
  a. At the end of the experiment, wait 5 min for the phases to separate in the microgel collection tube on ice (Figure 8A).

92. Mix 30 μL of cell-laden microgels from step 91e with 170 μL of 100% Matrigel.
93. Seed 50 μL of the mixture as a Matrigel dome (see details in^6^) in 24 well plate. If intended for imaging, seed 20 μL in a well of an IBIDI dish.
94. Leave for 15 min at 37°C and 5% CO_2_ incubator for Matrigel solidification
95. Overlay with cell culture media.
96. Monitor the growth of the culture over the next 7–10 days (Figure 9), changing the media every 3 days.

**CRITICAL:** Matrigel will solidify at 19°C–25°C, so work quickly and keep the basement matrix cold throughout the process.

**CRITICAL:** Prepare cell culture plates or dishes for seeding by pre-warming them in 37°C – at least 30 min in advance, but the plates can also be pre-warmed a day in advance.

## Expected outcomes

### Microfluidic chip production

Microfluidic chips should be clean and be devoid of any particles in the channels. The PDMS should be well attached to the microscopy slide and not detach upon operation. The inlets/outlets should fit with the desired tubing’s outer diameter, where the tubing can be fitted with ease whilst sitting there tightly (i.e., resistant to a mild pulling force). This will prevent the pressure of the flow and any unexpected strains from detaching the tubing. The chip should support a laminar flow of liquid, which can be tested with pure oil.

### Cell isolation and sorting

An expected yield for this part of the protocol is from 10 000 to 40 000 mesenchymal cells for one mouse, and 50 000 to 100 000 ductal cells. After seeding, mesenchymal cells are expected to attach within the first 3 days and show fibroblast morphology at passages 0–2, as illustrated in Figure 4.

### Liver portal mesenchyme *in vitro* culture:

By day 7–9 after seeding, the mesenchymal primary cells should be ready for passaging to passage 1. By day 14–16 they should be ready for passaging to passage 2. By 3 weeks from isolation, they should have expanded sufficiently for an encapsulation or co-culture experiment. Mesenchyme from 1–3 animals might be necessary for achieving enough cell numbers for subsequent microfluidic co-cultures. The 2D co-culture is more permissive to initial low isolation numbers. For the detailed analysis of gene expression at the time of cell isolation and during subculturing, please refer to Figures 2, Figure 4 and Figure S4 in ref. ^1^.

### Matrigel 2D layer co-culture:

In the first 3 days of the co-culture, and especially in higher ratios of mesenchyme to ductal cells (0.5:1 or above), large cell aggregates can be observed in brightfield and fluorescence microscope (Figure 5). The fully formed assembled 3D structures can be seen afterward, typically from day 4. At lower ratios of portal mesenchyme (below 0.5:1), formation of cystic ductal organoids should be observed, as shown in the most left panels of Figure 5 (no mesenchyme, or 0.1:1 ratio).

### Microfluidic encapsulation and co-culture

At the end of microfluidic encapsulation, formation of two phases should be apparent in the test tube used for collection: an oil phase (*bottom*) and an aqueous phase containing the cell-laden microgels (*top*) (Figure 8). The liquid agarose droplets should have solidified into microgels upon collection on ice. After the removal of the majority of oil and subsequent addition of media and PFO, the phases should be visibly separated as an oil and an aqueous phase, with a mixed layer in between the two phases (Figure 8). Microgels can be viewed on a brightfield or fluorescence microscope, if needed.

After 5–7 days in culture, the ductal organoids should be expanding and forming hollow cysts, as long as they do not have higher than 0.3:1 ratio of the mesenchyme in the structure (homeostatic ratio, as described in^1^). While the concentration of input cells is the same for both cell types, the encapsulation in the agarose microgel and therefore formation of the co-culture ratio is stochastic, occurring in any of the ratios from 0.1:1 to 10:1 (for details structure variety observed, please refer to Figure 5 and Figure S5 in ref. ^1^). If the ratio is higher, collapse of the organoid or the lack of growth is expected (Figure 9, Day 6, panel ii). The agarose microgels can be seen persisting in Matrigel co-culture and do not interfere with growth. In case of substantial organoid growth, the disintegration of agarose can be observed.

## Limitations

In this protocol, we describe co-cultures of primary cells: liver ductal cells and liver portal mesenchyme. However, it is quite challenging to retrieve more than 40 000 mesenchymal cells from one mouse liver - isolation of around 5000 cells is common. It is possible to scale the isolation up, combining several mouse livers, even though this significantly extends the sorting time. Additionally, we do not recommend expanding the mesenchyme more than 2 passages *in vitro*, as it becomes activated (similar to the activation of fibroblasts).

Another challenge remains controlling the stoichiometry of encapsulation in the microfluidic system, which at present is probabilistic (following a Poisson distribution) and can only be minimally adjusted by increasing or decreasing input cell density.

## Troubleshooting

### Problem 1

Low yield of liver mesenchymal cells (steps 1–49).

### Potential solution

It is usual to obtain low numbers of mesenchyme, typically 5000 cells, especially when digestion time has not been optimized before. To increase the number of sorted cells, altering the time of tissue digestion under the user’s specific conditions is recommended. Additionally, yield can vary between different batches of collagenase and dispase used. Yields above 40 000 cells from a single liver are rare, but possible.

### Problem 2

Low co-encapsulation efficiency (steps 84–96).

### Potential solution

The cause of this problem is usually low cell density in the starting material, below the 0.5–1 × 10^6^ cells/mL, specified in step 87d. Starting with higher number of isolated cells by increasing the number of mouse livers, or by expanding mesenchyme to achieve higher cell density is a potential solution.

### Problem 3

Clogging up of the microfluidic chip (steps 84–90).

### Potential solution

Clogging is a very frequent problem. Dust particles that block the microfluidic chip can originate from variety of sources. The most common source of blockage are fragments from the tubing or the chip itself. To avoid this issue, the tubing and the chip should be washed with the oil prior to the experiment. If the suspected source of dust particles is the cell suspension, additional cell washes after TrypLE treatment might be necessary to remove the debris (as described in step 60 for mesenchyme and 72 for ductal cells). If the suspected fraction is agarose, we recommend purchasing a new aliquot. Finally, if the dust particles appear to be originating from oil and/or surfactant, we recommend filtering those through a 0.22 μm filter.

### Problem 4

Low or no growth of cell co-cultures (steps 83 and 96).

### Potential solution

The most common issue for low co-culture growth is the use of old, expired media – the media should be prepared fresh and kept no longer than one month. The overall high ratios of mesenchyme to ductal cells in the culture can also cause low expansion of the co-culture, as the mesenchyme inhibits ductal growth through direct cell-cell contact.^1^ Also, if the overall numbers of mesenchyme in the culture well are low, the ductal cells do not expand well in MM media, which only contains WNT3a but otherwise is devoid of any of the growth factors normally present in the EM (expansion media), even if the ratio of mesenchyme to ductal cells is growth-permitting. Especially in the microfluidic encapsulation and subsequent co-culture, it is recommended in low-yield encapsulations to seed higher number of microgels per Matrigel dome, in order to increase the fraction of mesenchyme in the cell culture well.

### Problem 5

Dissociation/detaching of Matrigel in the co-cultures (steps 83 and 96).

### Potential solution

If the Matrigel becomes wobbly or unstable in the co-cultures, it can be caused by several factors, namely: lower than usual amount of total protein in the Matrigel, seeding Matrigel on a cold plate, or using cold media to put on top of Matrigel. To avoid these issues, Matrigel of known and constant protein concentration should be used; plates should be pre-warmed at 37°C before seeding cells with Matrigel; media should be pre-warmed at least until 19°C–25°C before pipetting it on the co-culture.

## Resource availability

### Lead contact

Further information and requests for resources and reagents should be directed to and will be fulfilled by the lead contact, Meritxell Huch (huch@mpi-cbg.de).

### Materials availability

This study did not generate new unique reagents.

## Data Availability

This study did not generate any code or analyzed any datasets.
